# Estimated direct and indirect health care costs of severe infectious keratitis by cultured organisms in Thailand: An 8-year retrospective study

**DOI:** 10.1371/journal.pone.0288442

**Published:** 2023-07-12

**Authors:** Somporn Chantra, Supachase Jittreprasert, Peranut Chotcomwongse, Anyarak Amornpetchsathaporn

**Affiliations:** 1 Department of Ophthalmology, Rajavithi Hospital, Bangkok, Thailand; 2 College of Medicine, Rangsit University, Bangkok, Thailand; Keimyung University, School of Medicine, Dongsan Medical Center, REPUBLIC OF KOREA

## Abstract

**Purpose:**

To evaluate the economic impact of treating severe infectious keratitis (IK) at one tertiary referral center in Thailand by analyzing the direct costs of treatment and estimating the indirect costs, and to determine whether cultured organisms had any effect on treatment expenditure.

**Methods:**

A retrospective study was conducted of patients with severe IK who had been hospitalized between January 2014 and December 2021 in Rajavithi Hospital. Data from medical records were collected from the time of the patients’ admission until the point at which they were discharged and treated in the outpatient department and their IK was completely healed, or until evisceration/enucleation was performed. The direct costs of treatment included fees for services, medical professionals and investigation, as well as for operative and non-operative treatment. The indirect costs consisted of patients’ loss of wages, and costs of travel and food.

**Results:**

A total of 335 patients were studied. The median direct, indirect and total costs were US$65.2, range US$ 6.5–1,119.1, US$314.5, range US$50.8–1,067.5, and US$426.1, range 57.5–1,971.5 respectively. There was no statistically significant difference between direct, indirect, or total treatment costs for culture-negative and culture-positive patients. Among those who were positive, fungal infections entailed the highest total cost of treatment, and this difference was statistically significant (p<0.001). In terms of direct and indirect costs, patients with fungal infections had the greatest direct costs, and this figure was statistically significant (p = 0.001); however, those with parasitic infections had the highest indirect treatment costs, and this was also statistically significant (p<0.001).

**Conclusion:**

Severe IK can cause serious vision impairment or blindness. Indirect costs represented the majority of the expense at 73.8%. There was no difference between direct, indirect, and total treatment costs for patients who were culture-negative or positive. Among the latter, fungal infections resulted in the highest total cost of treatment.

## Introduction

Infectious keratitis (IK) is a serious problem which can cause vision impairment or blindness [1, 2]. The Asia Cornea Society Infectious Keratitis Study (ACSIKS), a prospective non-randomized clinical multinational study, investigated final best-corrected visual acuity (BCVA) after IK treatment of 6489 eyes recruited from 11 study centers in Asia from March 2014 to November 2014. Moderate or worse visual impairment was found in 53.6% of study eyes [3]. Medical therapy is currently the primary treatment for IK; however, in some cases, this treatment alone is insufficient, and it is necessary to use surgical interventions such as therapeutic penetrating keratoplasty (TPK), lamellar keratoplasty, amniotic membrane transplantation, conjunctival flap transplantation, corneal gluing, evisceration, or enucleation [4]. The cost of treating IK with medication is high, and it is even greater if surgical intervention is required [5]. Approximately 14% of eyes with IK in the ACSIKS required surgical intervention, the most common being corneal transplantation, and it is unsurprising that about half of the grafts were ultimately failure [3]. As a result, IK not only has a negative impact on the patient’s quality of life but also places a significant burden on national health expenditure [6] with treatment costs varying by country. In Taiwan, a 14-year population-based study of the epidemiology and estimated burden of microbial keratitis on the health care system revealed that the average outpatient and inpatient expenditures for IK per episode were approximately US$71.94 and US$1,027, respectively [7]. In the UK, the median direct inpatient expenditure for IK was approximately US$3254.70 per patient [5]. Some studies have found that costs of IK treatment were higher for certain causative organisms [8], delayed onset of presentation [9, 10], and longer length of stay [5].

Kampitak K, et al., [8] a study from Thailand published in 2013, examined the direct costs of hospitalization, such as medicine, surgery, medical services, laboratory fees, and room charges. Some expenses may not be covered, such as direct costs of outpatient treatment after a patient is discharged from the hospital and requires follow-up in an outpatient department, indirect costs of treatment during hospitalization and follow-up in an outpatient department, such as lost wages, travel, and food costs. Understanding the cost of medical care for IK, especially severe forms that often require hospitalization, is an important aspect of a healthcare system’s financial planning. Therefore, the aim of this study was to evaluate the economic impact of treating severe IK at one tertiary referral center in Thailand, from the perspective of both patients and hospitals, by estimating the indirect costs of treatment and analyzing the direct health care costs. The other outcome was to determine whether the different types of cultured organisms had any effect on treatment expenditure.

## Material and methods

The study protocol followed the ethical guidelines regarding human subjects given by the Declaration of Helsinki and was approved by the Ethics Committee of Rajavithi Hospital (No. 65100) on 11 July 2022. The need for informed consent from participants was waived by the Ethics committee of Rajavithi Hospital due to the retrospective nature of our study. We included all patients presenting with severe IK who had been hospitalized in Rajavithi Hospital between January 2014 and December 2021. Each case in this study was classified as severe IK, according to modification of Jones’ grading criteria. Severe IK is defined by a diameter greater than 5 mm, a depth greater than 50%, dense infiltrates reaching the deep layers of the corneal stroma, and possibly scleral involvement [11]. This study used the electronic medical record audits in Rajavithi Hospital, and all cases were coded according to the International Classification of Diseases, 10^th^ Revision, Clinical Modification Codes (ICD-10-CM). The study included patients with a diagnosis of corneal ulcer (H160) who had been hospitalized with IK treatment as the primary indication. We excluded cases with insufficient data for evaluation as well as patients who had multiple hospitalizations during the same disease episode. We collected data on all eligible patients from the time they were admitted to the hospital to the time of their discharge and treatment in the outpatient department until the point when their IK was completely healed or evisceration/enucleation was performed. Corneal scraping was routinely taken from each patient’s affected eye upon admission to the hospital in order to culture the samples in blood, chocolate, and thioglycolate broth. In addition, PCR tests for herpes and non-nutrient agar with E. coli lawn were performed if there was suspicion of viral or parasitic infection. We defined healed IK as an epithelial defect that was completely closed, with all corneal infiltration having turned to corneal scar. When a therapeutic corneal transplantation was performed, final follow-up was considered when there was no epithelium defect and no recurrence of infection on the corneal graft. The following data were reviewed and analyzed: gender; age on admission date; duration of symptoms onset before presenting at our hospital; region of residence; length of stay (LOS); BCVA on first admission date and on final follow-up visit; duration of follow-up period; medical treatment; surgical intervention; number of visits to the outpatient clinic; and microbial culture results.

Thailand now provides universal health coverage to all Thais through one of three major public health insurance systems: the Civil Servants Medical Benefit Scheme (CSMBS), which provides medical coverage to government employees and their families, including parents; the national Social Security Scheme (SSS), which provides medical coverage to employees in the private sector; and the National Health Security Scheme, also known as the Universal Coverage System (UCS), which provides medical coverage to all Thais who are not members of the other two groups mentioned above [12]. The cost analysis in this study included expenses directly and indirectly attributable to patient care. The direct costs, costs directly related to patient care included service costs (room/food services); medical professionals fees (doctors’/nurses’/other health care professionals’ disbursements); investigation costs (lab/imaging/other tests); operative treatment expenses (surgical interventions such as intracameral injection, gluing, corneal biopsy, tarsorrhaphy, penetrating keratoplasty, lamellar keratoplasty, evisceration and enucleation/surgical instruments/anesthesia and related costs); and non-operative treatment outlay (pharmaceuticals/blood transfusion). We collected the direct healthcare costs which were paid to the medical institution by one of the major public health insurance systems, whether CSMBS, SSS, or UCS, depending on the patient’s health coverage. Indirect costs, those not directly related to patient care specified in the standard cost list for health and technology assessment in the Health Intervention and Technology Assessment Program (HITAP) website (https://costingmenu.hitap.net/), were patients’ loss of wages, travel expenses incurred in order to access care, and food expenditure. The estimated food costs and loss of wages were calculated using the number of days of work missed during hospitalization (LOS) and the number of hospital visits to the outpatient department following discharge. The estimated travel expenses incurred to access care were calculated by multiplying the standard cost of travel from the patient’s region of residence and the number of hospital visits. The cost data were converted to the year 2021 values using the Thailand Consumer Price Index (CPI) for medical care [13], and the adjusted figure was used for analysis. All costs were calculated in Thai Baht (THB) and then converted to US dollars (US$) from the 2021 exchange rate of 1 US$ = 35.0 THB in the year 2021.

### Statistical methods

Descriptive results were summarized for continuous variables using mean, median and range, and for categorical variables utilizing frequencies and percentages. Nonparametric testing with Kruskal Wallis test was employed for comparisons between groups of culture organisms, age on admission date, cost of treatment, gender, direct costs, and indirect costs. Statistical significance was set at p-value<0.05, and SPSS V.22.0 was used in all statistical analyses (IBM SPSS Statistics for Windows).

## Results

A total of 335 patients who had been admitted to the inpatient department were included, and their medical records from the inpatient and outpatient department relating to the same disease episode were analyzed. The majority of patients were male (214 patients, 63.9%). Age at presentation ranged from 7 to 88 years with a mean of 54.12 years (SD 17.7 years), and just under half (156 patients, 46.6%) were aged between 30 and 60 years. All patients had received topical medications from their primary/secondary care provider before presenting at our hospital. After classifying the infections based on clinical findings at presentation, out of the total 335 eyes revealed that 47.2% (158 eyes) were treated for bacterial infections, 37.9% (127 eyes) for fungal infections, 10.4% (35 eyes) for mixed bacterial and fungal infections, 2.7% (9 eyes) for viral infections, and only 1.8% (6 eyes) for parasitic infections. The classification of mixed bacterial and fungal infectious keratitis was based on the clinical history and findings of polymicrobial keratitis, which included the use of contact lenses, advanced age, the use of steroids, indistinct edges of the infiltrate, and the presence of ring infiltrate [14].

When cultured, 118 patients (35.2%) were found to have no growth for all organisms, 119 patients (35.5%) had positive culture for bacteria, 91 patients (27.2%) had fungal infections, 4 patients (1.2%) had positive culture for virus, 3 patients (0.9%) had parasitic infections which were all Acanthamoeba species. Table 1 shows the list of microorganism culture test results found in the samples.

**Table 1:** List of microorganism culture test results found in the samples.

Pathogens	Number of cases	Percentage of occurrence
**Bacterial**	119	35.5%
**Gram-negative**		
*Pseudomonas aeruginosa*	35	
*Staphylococcus hominis*	9	
*Staphylococcus haemolyticus*	4	
*Morganella morganii*	3	
*Klebsiella pneumoniae*	2	
*Moraxella species*	2	
*Acinetobacter baumannii*	1	
*Aeromonas hydrophila*	1	
*Burkholderia cepacia*	1	
*Neisseria gonorrhoeae*	1	
*Proteus mirabilis*	1	
*Vibrio cholerae*	1	
**Gram-positive**		
*Staphylococcus epidermidis*	17	
*Staphylococcus warneri*	7	
*Bacillus cereus*	6	
*Mycobacteroides abscessus*	5	
*Anaerobic Gram-positive bacillus*	3	
*Enterococcus faecium*	3	
*Coagulase-negative staphylococci*	3	
*Kocuria species*	2	
*Staphylococcus capitis*	2	
*Streptococcus pneumoniae*	2	
*Corynebacterium species*	1	
*Kocuria kristinae*	1	
*Propionibacterium acnes*	1	
*Paenibacillus species*	1	
*Streptococcus agalactiae*	1	
*Staphylococcus aureus*	1	
*Norcadia*	1	
*Mycobacterium tuberculosis*	1	
*Vibrio cholerae*	1	
**Fungal**	91	27.2%
**Mold**		
*Unidentified Mold species*	30	
*Aspergillus species*	26	
*Fusarium species*	24	
*Mucor species*	3	
*Rhizopus species*	2	
**Yeast**		
*Candida species*	5	
*Trichosporon asahii*	1	
**Viral**	4	1.2%
*Herpes simplex virus*	3	
*Epstein–Barr virus*	1	
**Parasite**	3	0.9%
*Acanthamoeba species*	3	
**Total**	**217**	**100%** *

* Calculated solely based on the number of patients with positive culture results

Prior to presenting at our hospital, patients’ median duration of symptom onset was 14.0 days (range 1–180 days). Median LOS was 15 days (range 1–74 days); median clinical visits after discharge from hospital until final follow-up of disease episode was 4 (range 1–16); and median treatment duration was 152 days (range 7–2328 days). At first presentation, 6 patients (1.8%) had vision better than 20/60, 18 (5.4%) had vision ranging from 20/60 to 20/200, 8 (2.4.%) had vision range worse than 20/200 to 20/400, 277 (82.7%) had vision worse than 20/400 to light perception (PL), and 26 patients (7.8%) had vision with no light perception in the affected eye. At final follow-up, 48 patients (14.3%) had vision better than 20/60, 28 (8.4%) had vision range from 20/60 to 20/200, 7 (2.1%) had vision range worse than 20/200 to 20/400, 149 (44.5%) had vision worse than 20/400 to PL, and 103 (30.7%) had vision with no light perception in the affected eye.

Almost half of the patients (162, 48.4%) did not receive TPK or evisceration/enucleation. Evisceration/enucleation was performed on 79 patients (23.5%) who had severe corneal infections that did not respond to treatment. TPK was performed on 101 patients (30.1%), while 7 individuals who had previously undergone TPK surgery required evisceration/enucleation due to recurrent infection on the corneal graft. The most common causative pathogen for evisceration/enucleation was bacteria (40 patients, 50.6%), but the most prevalent pathogen in patients who had evisceration/enucleation after previous therapeutic keratoplasty was fungus (5 cases, 71.4%). Eleven patients had undergone more than one TPK, and most of these had bacterial infections. The duration of symptom onset before presentation at the hospital, LOS, and number of follow-up visits according to cultured organisms. There was no statistically significant difference between these three factors in patients with negative and positive culture, p-value 0.432, 0.446, and 0.054 respectively. Among culture-positive patients, those with parasitic infections had the longest duration of onset of symptoms before hospital presentation, and this figure was statistically significant at p-value 0.004. Patients with fungal infection had the longest LOS among the culture-positive cases, and this was statistically significant at p-value 0.007. There was no significant difference between the number of follow-up visits required by culture-positive patients and their negative counterparts. Among the former, type of infection was not a significant factor. There was a statistically significant difference in the number of surgical treatments, BCVA at presentation, and BCVA at final follow-up visits between patients with negative and positive culture, with p-values of <0.001, <0.001, and <0.001, respectively. Table 2 shows the comparison of causative organisms from culture results showing number of patients, epidemiology, medical and surgical treatment, duration of symptoms onset before presentation at the hospital, LOS, number of follow-up visits, visual acuity distribution at presentation, and final follow-up.

**Table 2:** Comparison of causative organisms from culture results showing number of patients, epidemiology, medical and surgical treatment, duration of symptoms onset before presentation at the hospital, LOS, number of follow-up visits, visual acuity distribution at presentation, and final follow-up.

	Culture results						^a^p-value
	Negative	Positive					
		Bacterial	Fungal	Viral	Parasitic	^b^p-value	
**N**	118	119	91	4	3		
**Male (N, %)**	73	70	68	1	2	0.036*	0.514
**Age at presentation (years; mean ± SD)**	54.1±17.7	54.9±20.0	53.4±13.7	57.5±31.2	40.7±22.9	0.697	0.954
**Age group at presentation (N)**						0.341	0.821
<30 years	12	20	5	1	1		
30–60 years	57	41	55	1	2		
>60 years	49	58	31	2	0		
**Number of surgical treatments (N)**						0.138*	<0.001*
1 TPK	33	18	41	0	0		
> 1 TPK	0	6	3	1	0		
evisceration/ enucleation	15	40	23	1	0		
**Duration of symptoms onset (day; median, min-max)**	14.0 (1.0–120.0)	7.0 (1.0–90.0)	14.0 (1.0–180.0)	14.0 (7.0–150.0)	30.0 (7.0–30.0)	0.004*	0.432
**LOS (day; median, min-max)**	14.0 (1.0–60.0)	14.0 (1.0–74.0)	21.0 (3.0–73.0)	11.0 (6.0–77.0)	16.0 (14.0–29.0)	0.007*	0.446
**Number of follow-up visits (median, min-max)**	4 (1–16)	4 (1–16)	5 (1–10)	5 (2–11)	6 (5–11)	0.083	0.054
**BCVA at presentation (N)**						0.004*	<0.001*
< 20/60	4	1	1	0	0		
20/60-20/200	11	1	6	0	0		
< 20/200-20/400	4	2	2	0	0		
<20/400-PL	97	94	79	4	3		
NPL	2	21	3	0	0		
**BCVA at final follow-up (N)**						0.214	<0.001*
< 20/60	25	10	12	1	0		
20/60-20/200	14	7	6	1	0		
< 20/200-20/400	3	2	2	0	0		
<20/400-PL	58	47	40	1	3		
NPL	18	53	31	1	0		

^a^ p-values were calculated using Mann-Whitney U Test between culture-positive and culture-negative patients.

^b^ p-values were calculated using Kruskal Wallis test among culture-positive patients.

* = significant p-value <0.05

N, number of patient(s); TPK, therapeutic keratoplasty; BCVA, best corrected visual acuity; PL, light perception; NPL, no light perception

The median direct cost was US$65.2, range US$ 6.5–1,119.1 (mean US$140.1, SD US$184.8). Median indirect cost was US$314.5, range US$50.8–1,067.5 (mean US$7,355.2 SD US$189.7), and median total cost was US$426.1, range US$57.5–1,971.5 (mean US$495.3, SD US$312.6). Among all age groups, people in the age range 30–60 years old had the highest direct, indirect, and overall total cost of treating severe IK, and these figures were statistically significant (p-value 0.006, 0.033, and 0.004 respectively). There was no statistically significant difference between males and females in terms of direct and indirect costs (p-value 0.179 and 0.068), but overall total cost was significantly higher in males than in females with p-value 0.035.

Table 3 shows the direct costs of treatment of inpatients and outpatients according to culture organism. There was no statistically significant difference (p-value 0.234) between total direct treatment costs for patients with negative (median US$62.9, range US$6.5–935.9) and positive cultures (median US$66.3, range US$6.6–1,119.1), regardless of whether the costs were incurred as part of inpatient or outpatient care, p-value 0.259 and 0.423 respectively. Among culture-positive patients, cases of fungal infections resulted in the highest expenditure for inpatient care, primarily in terms of services, non-operative care, and total direct inpatient care costs, with each of these being statistically significant at p-value 0.024, <0.001, and <0.001 respectively. Of culture-positive cases, those with parasitic infections had the highest outpatients care costs, and this was statistically significant (p-value 0.04).

**Table 3:** Comparison of direct costs of treatment of severe IK for inpatients and outpatients according to causative organism.

		Culture results						
		Negative N = 118	Positive					^a^p-value
			Bacterial N = 119	Fungal N = 91	Viral N = 4	Parasitic N = 3	^b^p-value	
**Inpatient care costs**	**Services costs**	1.9 (0.1–122.8)	2.1 (0.1–168.4)	4.0 (0.1–302.2)	1.8 (0.6–8.6)	3.5 (2.5–36.2)	0.024*	
	**Investigation costs**	3.2 (0.3–43.4)	3.6 (0.4–40.2)	5.7 (0.3–33.6)	3.02 (1.8–4.2)	5.1 (2.6–7.7)	0.856	
	**Operative costs**	1.66 (0.1–218.7)	2.2 (0.1–606.9)	5.5 (0.1–405.2)	1.4 (1.2–30.9)	4.6 (2.8–20.6)	0.636	
	**Non- operative costs**	18.2 (0.0–751.6)	15.3 (0.0–728.7)	65.0 (0.9–555.7)	17.7 (7.9–79.2)	28.2 (11.2–90.9)	<0.001*	
	**Total costs**	28.4 (0.4–910.9)	20.8 (0.5–1062.7)	76.8 (2.8–976.2)	20.9 (11.7–123.0)	36.3 (19.2–155.5)	<0.001*	0.259
**Outpatients care costs**		24.9 (6.1–98.7)	25.0 (6.1–97.8)	30.5 (6.1–69.9)	27.6 (12.4–42.8)	36.6 (31.0–76.9)	0.04*	0.423
**Total direct costs**		62.9 (6.5–935.9)	52.5 (6.6–1119.1)	120.7 (9.1–1046.1)	57.7 (24.1–147.5)	67.3 (55.9–232.4)	0.001*	0.234

^a^ p-values were calculated using Mann-Whitney U Test between culture-positive and culture-negative patients.

^b^ p-values were calculated using Kruskal-Wallis test among culture-positive patients.

* = significant p-value <0.05

N = number of patient(s)

Values are presented in US$ as median (minimum-maximum)

Table 4 analyses the indirect costs incurred in managing severe IK during hospitalization and follow-up in the outpatient department according to causative organisms. There was no statistically significant difference (p-value 0.26) between total indirect treatment disbursements for culture-negative patients (median US$287.6, range US$50.8–912.1) and those with positive culture (median US$319.8, range US$75.7–1067.5) regardless of whether the costs were incurred during hospitalization or follow-up in the outpatient department, p-value 0.423 and 0.053 respectively. Of the culture-positive cases, those with parasitic infections had the highest total indirect costs, and this was statistically significant (p-value<0.001). There was no statistically significant difference between loss of wages, food costs, or travel costs incurred by patients with different cultured organisms during the follow-up period in the outpatient department. Of patients with positive culture, those with fungal infection had the highest indirect cost during hospitalization (p-value 0.005).

**Table 4:** Indirect costs of treatment (loss of wages, travel and food costs) during hospitalization and follow-up in the outpatient department according to severe IK culprit organisms.

		Culture results						
		Negative N = 118	Positive					^a^p-value
			Bacterial N = 119	Fungal N = 91	Viral N = 4	Parasitic N = 3	^b^p-value	
**During hospitalization**	**Patients’ lost wages**	169.1 (12.0–725.0)	166.6 (12.0–1007.2)	258.3 (36.5–876.9)	102.1 (71.4–396.4)	193.3 (190.5–34.2)	0.005*	0.423
**During follow-up period in outpatient department**	**Patients’ lost wages**	48.5 (11.9–192.2)	48.7 (12.0–190.4)	59.5 (11.9–136.1)	53.8 (24.1–83.3)	71.4 (60.4–149.7)	0.064	
	**Travel costs**	44.75 (10.9–177.1)	44.9 (11.0–175.5)	54.8 (10.9–125.4)	49.6 (22.2–76.8)	65.8 (55.6–138.0)	0.064	
	**Food costs**	14.0 (3.4–55.7)	14.1 (3.4–55.2)	17.2 (3.4–39.4)	15.6 (7.0–24.1)	20.7 (17.5–43.4)	0.064	
	**Total**	107.3 (26.3–425.1)	107.8 (26.5–421.3)	131.6 (26.3–301.0)	119.0 (53.4–184.3)	157.9 (133.6–331.1)	0.064	0.053
**Total indirect costs**		287.6 (50.8–912.1)	286.6 (75.7–1067.5)	399.7 (85.8-1003-1)	221.0 (176.7–529.3)	503.2 (326.9–521.7)	<0.001*	0.260

^a^ p-values were calculated using Mann-Whitney U Test between culture-positive and culture-negative patients.

^b^ p-values were calculated using Kruskal-Wallis test among culture-positive patients.

* = significant p-value <0.05

N = number of patient(s)

Values are presented in US$ as median (minimum-maximum)

Table 5 shows an analysis of direct, indirect, and total costs of treating severe IK per episode/per patient based on initial and final BCVA. From Kruskal Wallis test, the direct, indirect, and overall total costs were significantly different for each visual acuity group, and patients with BCVA presentations of less than 20/400 to PL incurred the greatest expense in all three cost areas.

**Table 5:** The direct, indirect, and overall total costs of treating severe IK per episode/per patient based on initial and final BCVA.

	Total direct costs		Total indirect costs		Overall total costs	
	Median	Min-Max	Median	Min-Max	Median	Min-Max
**BCVA at presentation**						
< 20/60	68.0	9.9–1046.1	155.3	139.0–654.9	286.9	148.9–1701.1
20/60-20/200	46.5	6.6–466.1	177.6	50.8–471.7	226.0	57.5-937-9
< 20/200-20/400	55.1	6.6–501.8	304.3	151.8–646.9	453.9	178.3–1093.0
<20/400-PL	74.6	6.5–1119.1	351.0	73.9–1067.5	474.3	81.1–1971.5
NPL	36.2	6.6–842.7	280.3	112.1–321.8	248.1	127.0–1111.7
p-value ^a^	0.012*		<0.001*		<0.001*	
**BCVA at final follow-up**						
< 20/60	65.3	6.9–1046.1	391.2	94.8–2631.1	1997.8	169.8–33971.6
20/60-20/200	62.5	6.6–905.0	457.2	73.3–1651.6	2324.9	326.1–29174.4
< 20/200-20/400	42.5	8.5–117.1	367.0	116.6–2055.0	1198.0	230.2–4275.7
<20/400-PL	86.8	6.6–1119.1	576.1	62.6–2621.5	2885.7*	120.1–39707.5
NPL	50.5	6.5–842.7	559.0	88.5–3168.9	1760.1	204.4–29289.5
p-value ^a^	0.068		0.005*		0.018*	

Values are presented in US$ as median (minimum-maximum)

* = significant p-value <0.05

^a^ p-values were calculated using Kruskal Wallis test among positive cultured patients

BCVA, best corrected visual acuity

PL, light perception

NPL, no light perception

## Discussion

This study is the first to analyze the direct and indirect costs of treating severe IK patients in Thailand who require hospitalization. As a secondary objective, it aimed to determine whether type of organism had any effect on treatment costs. In our research, more than half of the patients were male, with a median age of 55 years (range 7–88 years), which is similar to that of a previous ACSIKS study of subjects in 8 Asian countries, including Thailand, which found that the majority of IK patients were male, with a median age of 46.0 years (range 0.08–95 years) [3]. Using an age classification of under 30, 30–60, and over 60 years old, we found that the age range of 30 to 60 years old incurred the highest direct, indirect, and overall treatment costs. This could be attributable to the high prevalence of IK in this age group and their increased exposure to risk factors that could lead to the development of the disease. This finding is consistent with those of a review of 14 epidemiologic studies from Asia, the United Kingdom and Europe, North America, South America, Africa, the Middle East, and Australia, which revealed that the majority of IK cases were people between the ages of 30 and 55, and that use of contact lenses and ocular trauma were primarily responsible for IK’s high prevalence in this working-age range [15].

A review of 20 studies found that the overall median culture positivity rate from clinically diagnosed cases of IK was 50.3%. (range 32.6%–79.4%) [16]. Even though all our patients had previously received topical treatment from their primary/secondary care provider before coming to us, the culture positivity rate for IK was 65.2%, which is relatively high. In the culture-positive patients, the most common infection types were bacteria at 35.5%, followed by fungus at 27.1%, and this was consistent with results of previous studies, including some from Thailand [3, 17]. The number of TPK was found to be higher in culture-positive patients than in culture-negative patients in our study. A number of factors could account for this. Firstly, some microorganisms in culture-positive IK, particularly fungal IK, which constitutes the majority of patients in our study, are typically more virulent or resistant to treatment, complicating management and necessitating more aggressive treatment strategies such as TPK to control the infection and preserve vision [18, 19]. Secondly, as determined by BCVA at presentation, culture-positive patients were significantly more clinically severe than culture-negative patients in our study. In such situations, when the infection has reached an advanced stage where medical treatment is no longer effective, TPK may be the only option to preserve the eye, while evisceration or enucleation may be the sole treatment option available.

Some studies have found differences in BCVA between patients with culture-positive and culture-negative IK at presentation. The study by Ting DSJ et al. [20], which was conducted in the United Kingdom and included 283 patients of IK, found that culture-positive patients had statistically significantly worse BCVA at presentation compared to culture-negative patients (p<0.001). This finding was consistent with our study, which found that BCVA at presentation was statistically significantly worse in culture-positive patients than in culture-negative patients (p-value 0.001). However, some studies have found differences in BCVA at presentation between patients with culture-positive and culture-negative IK, but no statistically significant difference [21, 22]. In the study by Bhadange et al. [22], which included 60 patients with IK, the mean BCVA at the last follow-up was 1.83 LogMAR for culture-positive IK and 2.34 LogMAR at presentation for culture-negative IK. However, the final BCVA at the last follow-up was not statistically significant (p = 0.07) between the culture-positive and culture-negative groups. These results suggest a trend towards worse visual outcomes in culture-positive IK, but the difference did not reach statistical significance in their study. Yarimada et al. [21], in their study of 314 IK patients, found no statistically significant difference in BCVA between culture-positive and culture-negative patients at the end of the follow-up (p = 0.716). The mean BCVA for culture-positive patients was 1.21 ± 1.30 LogMAR, while it was 1.27 ± 1.29 LogMAR for culture-negative patients. However, our study, which included 335 patients, demonstrated a statistically significant difference in BCVA between culture-positive and culture-negative patients at the final follow-up (p-value 0.001). This suggests that culture-positive patients had worse visual outcomes compared to culture-negative patients. It is important to consider that visual outcomes in IK are influenced by multiple factors, including BCVA at presentation, severity of infection, type of microorganism involved, and the presence of comorbidities [15, 20].

Other studies have been conducted to assess the economic impact of IK [5, 8, 9, 23, 24]. Ashfaq H, et al. [23] focused on procedures, visits, and procedure costs in management of IK from a hospital perspective in the USA. On average, 2.9 (SD 4.2) interventions were performed per patient, resulting in a total cost of US$1,788.7 (SD US$3,324.62). According to Moussa, G. et al. [5], the median direct cost of inpatient care for treating IK in the UK was £2855 (IQR £2,018–4,057), which is equivalent to approximately US$3,254.7 using the 2020 average exchange rate of £1 = US$1.14. Prajna VN et al. [9] performed research in South India and calculated that the mean total cost of treating IK, including both direct and indirect costs, was US$85.5 (SD US$4.6, 95% CI: US$76.4, 94.6). Kampitak K, et al. [8], in a study of 53 patients in Thailand conducted in 2013, reported that the median direct cost of admission of IK was 20,699.0 THB (IQR 11,379.0–56,981.0), which is equivalent to approximately US$637.7 using the 2013 average exchange rate of 1US$ = 30.72 baht in 2013. Our study had lower direct health care costs when compared to Kampitak K, et al. study [8], which could be attributed to the causative organisms. In their study, nearly half of the cases had no growth for all organisms (45.3%), fungus 28.3%, bacteria 26.4%, whereas in our study, approximately one-third of the cases (35.2%) had no growth for all organisms, bacteria 35.5%, fungus 27.2%, virus 1.2%, and 0.9% had parasite infection. As we all know, culture-negative keratitis is a significant management issue [25]. In general, the initial treatment of IK is based on the patient’s history and clinical findings at the time of presentation. After that, treatment should be modified based on the results of culture and susceptibility testing [26]. The results of cultures are often helpful in selecting the appropriate treatment. In culture-negative IK, treatment can be lengthy, characterized by multiple referrals, changing treatment regimens, inconclusive investigations, and the need for additional diagnostic tests [25]. These factors may result in higher treatment costs. In our study, the median direct cost of treating one episode of severe keratitis was US$65.2, range US$6.5–1,119.1 (mean US$140.1, SD US$184.8), with no difference between direct costs for culture-negative and culture-positive cases. Among the latter, those with fungal infections had the highest direct costs, owing primarily to inpatient care costs, mostly non-operative costs, during hospitalization. Non-operative expenses in the inpatient department were chiefly driven by disbursements on antifungal treatments used, such as topical Natamycin and azole medications. The following are some common antifungal medications in Thailand, along with price information from Thailand’s National List of Essential Medicines [27]. Amphotericin B powder 50 mg costs THB176.55 (approximately US$5.04) for the preparation of 0.15% topical amphotericin B 33 ml. The cost of voriconazole powder 220 mg is THB3,779.78 (approximately US$107.99) for a supply of 1% topical voriconazole 20 ml. The cost of 0.1% Natamycin 15 ml is THB2,568 (approximately US$73.37). A study by Radhakrishnan N, et al. also found that total medication expenses were significantly greater in fungal keratitis than in the bacterial type, and they attributed this to the higher prices of antifungals compared to those of antibacterial medications [28]. Patients with parasitic infections incurred the highest direct outpatient care cost, and this was most probably due to the longer duration of medications needed to control these infections in comparison to antiamoebic medications, such as polyhexamethylene biguanide or chlorhexidine. All parasite cultures in this study were of the Acanthamoeba species, and Acanthamoeba keratitis is known to be difficult to treat due to the resilient nature of its cyst form. The length of time required for Acanthamoeba clearance from the cornea may vary from 3.25 to 26 months [29] whereas fungal keratitis treatment is shorter, at an average of 3–4 weeks [30].

The median indirect cost of having one episode of severe keratitis was US$314.5, range US$50.8–1,067.5 (mean US$7,355.2 SD US$189.7). There was no difference between indirect costs for patients with negative and positive culture. Among culture-positive patients, parasite infections led to the highest median total indirect cost, primarily resulting from costs incurred during the follow-up period in the outpatient department. Patients with parasite infection had the highest median costs in all categories during the follow-up period in the outpatient department, even though it was not statistically significant in each category. Of the culture-positive cases, those with fungal infections incurred the highest median indirect inpatient care cost. The main financial factor was LOS, probably because patients with fungal infections had the highest LOS in our study (21 days, range 3–73 days). This finding was consistent with those of a study in the UK, where the LOS for fungal keratitis was found to be 18.9 days (SD 16.3 days) [31], which was longer than for bacterial keratitis. Wong T, et al. [32] found that treatment of severe bacterial keratitis in New Zealand involved a mean hospital LOS of 5.8 days, with a median of 4.0 days, (IQR 2.0–8.0 days) while another study from UK, the Nottingham Infectious Keratitis Study, found that mean LOS was 8.0 days (SD 8.3 days) for hospitalized patients with bacterial keratitis [20]. In our study, the median LOS for bacterial keratitis was 14 days (range 1–74 days), and this longer duration may be due to our lack of cornea specialists in remote areas of Thailand, which means that we are unable to refer patients back to primary or secondary care and have to admit them to our tertiary care institutes for extended stays. A previous study has shown that LOS is the major factor influencing the direct cost of admitted IK cases [5]. To lower LOS, it is recommended that hospitals encourage the discharge of patients who are able to self-administer treatment following the sterilization phase, improve workflow, and increase cross-disciplinary interaction between ophthalmologists, microbiologists, pharmacists and nursing staff [5, 33]. Teleophthalmology and artificial intelligence could play a role in monitoring patients at home with the help of a health care provider after a hospital stay when they are partially recovering from a disease, in order to obviate the need for patients to travel to the hospital [34, 35].

Besides the burden placed on patients by their loss of wages during hospitalization, prolonged LOS is also a drain on hospital resources. The number of hospitalizations is an important factor when calculating direct costs such as service and medical professionals’ disbursements. When assessing the indirect cost of patients’ loss of wages, the LOS and number of hospital visits during the follow-up period were significant multipliers; therefore, reductions in these two areas are important factors in lowering the financial burden shouldered by patients. However, indirect cost is thought to be a factor that is determined by the patient’s residential area, occupation, and socioeconomic circumstances rather than disease characteristics. As a result, each patient’s actual expense would be more detailed and represent the actual indirect costs of severe IK treatment rather than the average standard indirect costs from HITAP. This is additionally one of the important considerations.

Our results indicate that patients with lower initial BCVA at presentation tend to have higher treatment costs, with cases of initial BCVA of less than 20/400 to PL incurring the highest direct, indirect, and overall costs. Patients with initial BCVA of NPL had the lowest treatment costs in this study; however, this does not necessarily imply an improvement in their IK. It is more probably attributable to the fact that these patients had severe infections and required evisceration/enucleation early in their treatment, resulting in a shorter LOS (median LOS of patients who had evisceration/enucleation was 9 days, range 5–20 days), and also fewer medications, surgical interventions, and follow-up visits. The BCVA at initial and final follow-up, together with the costs associated with each BCVA group level, provides us with an insight into the costs associated with treating every disease episode of severe IK in each BCVA category. The majority of patients in the study had initial and final BCVA at follow-up of 20/400 to PL, and this group had the highest treatment costs; however, the treatment effort and costs did not improve the patients’ BCVA, but rather merely maintained their level of vision.

The median total cost was US$426.1, range US$57.5–1,971.5 (mean US$495.3, SD US$312.6), with indirect costs representing by far the largest proportion of expenditure at 73.8%. There was no difference between total costs incurred by patients with negative and positive cultures, but among the latter, those with fungal cultures accounted for the highest total cost (median US$576.7, range US$100.1–1701.1). The World Data Atlas shows that annual health expenditure per capita in Thailand was US$296 in 2019 [36]. This means that the direct cost of treatment of one episode of severe IK, may exceed the average expenditure on health care for the entire year in some cases.

The data also show that Thailand’s general government health expenditure to current health expenditure ratio was 71.7%. This reflects the government’s primary concern regarding controlling healthcare costs, particularly by reducing the incidence of preventable diseases such as IK. Increasing public awareness and knowledge of the risk factors, early signs, and symptoms of IK is essential, as is encouraging the wearing of safety equipment while working in industry, driving, doing construction work, gardening, or engaging in sport. Other desirable measures include informing people how to properly wear and care for contact lenses, developing and effectively implementing teleophthalmology between regional and local health care systems, as well as between medical professionals and patients, in order to aid in early diagnosis, timely treatment, and monitoring of diseases. All of these interventions may result in lower overall healthcare costs throughout the treatment process.

This economic burden study was solely focused on treating episodes of severe IK and did not take into account the concomitant consequences of patients’ impaired vision, such as depression, which should not be overlooked. Rasendran C, et al. [37] showed that ophthalmic patients with depression presented an increased economic expense of US$5,894.8 per patient annually. Besides the economic impact, attendant human factors which include patient’s health-related quality of life, activities of daily living, as well as care givers’ health and quality of life, should also be considered.

## Limitations

This study has some limitations. First, the data were collected in a single tertiary care hospital in the central region of Thailand, so the findings are not representative of hospitals in other parts of the country. Since all these patients were referred from elsewhere after failing to respond to medications, they were more likely to have complicated and severe conditions that required additional management, potentially resulting in an overestimation of normal expenditure in our study. Second, we focused on patients with severe IK only and did not attempt to reflect the cost of treatment for all levels of severity. Clearly, mild and moderate cases of IK would be less expensive to manage because there would be no hospitalization and little surgical intervention required, so that the average cost of treatment of IK of other severity levels might be lower. Third, the indirect costs in this study were estimations rather than actual expenses because they were derived from the HITAP website’s average standard cost list rather than directly from each patient, and the loss of wages of accompanying persons was not calculated as part of the indirect care cost because the number of accompanying persons was not recorded. Fourth, 17 patients who had multiple hospitalizations during the same IK episode were excluded from the study. Such patients with multiple hospitalizations frequently have complicated diseases requiring more investigation and treatment than usual; therefore, the estimated cost of treatment in this study might be underestimated.

## Conclusion

Severe IK can cause serious vision impairment or blindness. This study provides information on the economic burden of treating severe IK in Thailand. There was no difference between direct, indirect, and total costs of treatment of severe IK for patients with negative or positive culture; however, among the culture-positive cases, fungal infections entailed the highest total cost of treatment.
